# Impact of bicarbonate on ionized calcium and cardiac function in albumin‐free Langendorff perfusion in hearts of male rats

**DOI:** 10.14814/phy2.71053

**Published:** 2026-08-10

**Authors:** Jan Doul, Zuzana Charvátová, Martin Modrák, David Kala, Jakub Otáhal

**Affiliations:** ^1^ Department of Pathophysiology, Second Faculty of Medicine Charles University Prague Czech Republic; ^2^ Department of Bioinformatics, Second Faculty of Medicine Charles University Prague Czech Republic

**Keywords:** bicarbonate, calcium, ionic activity, ischemia, Krebs–Henseleit, preload

## Abstract

The adult Langendorff methodology lacks standardization, with inconsistencies in the pH and calcium concentrations of Krebs–Henseleit (K‐H) solution. The aim of this study was to clarify these inconsistencies. Adult male Wistar rat hearts were subjected to 40 or 80 min of global ischemia followed by reperfusion. Recovery of left ventricular developed pressure (LVDP) and triphenyltetrazolium (TTC) staining were evaluated. Ion analysis was performed using rat plasma. A calcium concentration of 1.75 mM was associated with increased contractile force and more severe ischemia–reperfusion injury. The absence of preload resulted in increased variability in the results. LVDP recoveries were high and decreased with longer lengths of ischemia. Rat plasma exhibited higher total calcium and lower albumin levels than humans. The K‐H solution forms substantial amount of calcium complexes with bicarbonate, likely due to hypoalbuminemia, which also explains its alkaline nature. Rat plasma calcium activity closely corresponded to the K‐H solution with 1.75 mM Ca^2+^. These findings indicate that Langendorff perfusion experiments benefit from higher calcium and lower bicarbonate concentrations. Caution is warranted when preparing artificial solutions lacking albumin, as physiological ion concentrations may not correspond to physiological ion activities. Use of preload and longer lengths of ischemia may further improve experimental consistency.

## 1 INTRODUCTION

To investigate the mechanisms of ischemia–reperfusion (I/R) injury, isolated heart perfused according to Langendorff is a frequently used model. This model allows to examine the extent of I/R injury and to apply cardioprotective interventions, such as ischemic preconditioning (IPC), ischemic postconditioning (IPoC) or pharmacological protections (Lim et al., 2007). It also allows studying individual mechanisms of cardioprotective interventions by applying chemical substances that affect I/R injury or cardioprotective interventions (such as kinase blockers (Mittal et al., 2016), nitric oxide (Doul et al., 2024), substances affecting calcium overload (Zhang et al., 2006) or substances affecting mitochondrial permeability transition pore (mPTP) (Gonzalez Arbelaez et al., 2016)) while monitoring the outcomes of such intervention.

However, the methodology of adult Langendorff is not unified among different researchers. Studies vary in a large number of parameters, including use of anesthesia (or lack of it) (Fenton et al., 2005; Ferko et al., 2018; Inserte et al., 2008; Moheimani et al., 2021; Reichelt et al., 2009), constant pressure vs. constant flow system (Correa et al., 2008) or individual perfusion pressure values (Inserte et al., 2008; Moheimani et al., 2021), electrical pacing of the heart (or frequently a lack of it) (Fenton et al., 2005; Gonzalez Arbelaez et al., 2016; Moheimani et al., 2021), local vs. global ischemia (Babiker et al., 2019; Horvath et al., 2021) and the length of ischemia (Babiker et al., 2019; Gonzalez Arbelaez et al., 2016; Venditti et al., 2011). These variables can potentially influence the outcome of Langendorff studies (see recent review (King et al., 2022)). Type of anesthesia also has an effect on cardioprotection (many anesthetics clearly show cardioprotective properties (Ma et al., 2013, Shirakawa et al., 2014, Sloan et al., 2011)), while other anesthetics (pentobarbital) influence calcium metabolism (Altura et al., 1980; Nayler & Szeto, 1972).

Another potential source of variability is the strain and sex of rats used in the experiment. Most preclinical experiments are conducted on healthy strains of both sexes. It is generally accepted that the female sex is cardioprotective due to the effect of estrogens (Lagranha et al., 2010; Simoncini et al., 2000), although some studies challenge this (Lieder et al., 2019). The experiments also do not take into account the fact that most patients are older, postmenopausal and with multiple comorbidities. All of these discrepancies may be the reason for the long‐term failure to transfer the results of experimental studies related to cardioprotection to clinical medicine (Cung et al., 2015; Hausenloy et al., 2019).

To determine the extent of I/R injury, various methods can be used. The most common approach is the TTC (Triphenyl tetrazolium chloride) staining of the slices of the heart, based on marking only viable regions (with vital mitochondria) and comparison against “area at risk”, which is determined by staining the perfused myocardium with another dye (Evans Blue) (Wensley et al., 2013) in local ischemia. Experiments using global ischemia typically consider the entire slice (or entire LV area) to be the “area at risk” (Fenton et al., 2005; Kindernay et al., 2021). Other methods include the percentage recovery of a force of contraction measured by having an inflated balloon inside the ventricles and comparing a force of contraction before and after the ischemia (Reichelt et al., 2009). Another technique used, for example, in neonatal studies (Doul et al., 2024), is the measurement of the force of contraction using a force transducer attached to the apex of the heart with a suture. This method is considered to be less accurate, as it measures the force in only one direction. Rate‐pressure product has also been used in the past, though its use is somewhat criticized (Aksentijevic et al., 2016). Finally, to evaluate the extent of I/R injury, the release of enzymes from the heart can be used. The advantage of Langendorff's ex vivo studies is that less specific enzymes than troponins, such as LDH, can also be used (Ferko et al., 2018). Unfortunately, a number of studies including ours (Doul et al., 2015; Piot et al., 2008) described large variance and therefore low accuracy of these results.

The composition and biochemical properties of the perfusion solution can have a significant impact on the cardioprotection. Solution used to perfuse the heart is usually the Krebs–Henseleit (K‐H) solution saturated with 95% O_2_ and 5% CO_2_ gas mixture. Many variations in K‐H solutions have been used in the past. Notably, studies differ in the amount of calcium used (~1.25 mM (Gonzalez Arbelaez et al., 2016, Horvath et al., 2021, Paulova et al., 2013); ~1,75 mM (Hausenloy et al., 2007; Inserte et al., 2008); ~2–2,5 mM (Fenton et al., 2005; Reichelt et al., 2009)), bicarbonate used (20 mM (Fantinelli et al., 2013, Gonzalez Arbelaez et al., 2016); 22 mM (Reichelt et al., 2009); 24–25 mM (Hausenloy et al., 2007, Inserte et al., 2008)) and glucose used (<=7 mM (Doul et al., 2024, Ferko et al., 2018); ~11 mM (Horvath et al., 2021; Torregroza et al., 2021)). Some studies use higher glucose as an easy way to compensate osmolality due to other compounds not present in K‐H solution. However, hyperglycemia itself is known to have a negative impact on cardioprotection (Torregroza et al., 2021). Rats have approximately the same glycemic values as humans (Zhang et al., 2003) and therefore the use of mannitol is preferable in our opinion (Doul et al., 2024).

Calcium is known to affect I/R injury and a reduction of calcium levels (Reichelt et al., 2009), or a blockade of individual components of calcium overload alone is sufficient to protect the heart from I/R injury. These components probably include membrane Na^+^/Ca^2+^ exchanger (NCX) (Kaljusto et al., 2010), SERCA (Shintani‐Ishida et al., 2012) and a mitochondrial calcium uniporter (MCU) (Cao et al., 2006; Zhang et al., 2006). Mice lacking MCU are protected from the I/R injury (Luongo et al., 2015). For a review on a role of calcium in I/R injury, see (Rizzuto et al., 2009). Reichelt et al. (Reichelt et al., 2009) demonstrated in a mouse study that greater contractile force and greater I/R injury were observed in the free calcium concentration range of 1.5–2.0 mM. Calcium 1.8 mM was also used while studying arrhythmias (Ghais et al., 2009). However, these values are higher than would be expected under in vivo conditions, where free (ionized active) calcium accounts for about half of the total calcium and should correspond to a concentration of approximately 1.25 mM.

Similarly, pH plays a role in cardioprotection. Acidotic reperfusion is protective on its own (Inserte et al., 2008) while alkaline reperfusion negates the effect of the cardioprotective interventions (Hausenloy et al., 2007). A mechanism linking pH and calcium overload has been proposed (see (Ovize et al., 2010)). Combined action of NCX and NHX (Na^+^/H^+^) exchanger leads to proton current eventually translating into calcium current, thus alleviating or aggravating calcium overload. Additionally, acidosis has been shown to activate cardioprotective kinases (Cohen et al., 2007). According to the Hendersson‐Hasselbalch equation, we would assume the use of normal bicarbonate (~25 mM) in the perfusate. Rats appear to have normal pH and normal or higher bicarbonate levels (approximately 25–30 mM, calculated by the Hendersson‐Hasselbalch equation from the data in this study (Svorc et al., 2018)), however, some studies, without adequate explanation, have reported that reduced bicarbonate levels (20 mM) are necessary to achieve a normal pH in the K‐H solution (Fantinelli et al., 2013; Gonzalez Arbelaez et al., 2016).

The potential choice of sympathomimetic added to the perfusate is also important. Crucially, cAMP pathway is closely linked to intracellular calcium signaling (Schmid et al., 2014; Taylor, 2017). This prompted the question of whether the requirement for higher calcium in the Langendorff heart might be influenced by the lack of sympathetic innervation (i.e. whether calcium‐dependent effects are partly unmasked in the absence of sympathetic tone). It has been previously reported that dobutamine has no effect on I/R injury (Ooiwa et al., 1989) and increased heart rate does not worsen I/R injury (Reichelt et al., 2009) (surprising in a state of calcium overload with increased energy expenditure). As a result of vasoconstriction in the coronary arteries, a decrease in calcium in the perfusate at the onset of cardioprotection may also occur (probably reducing the calcium overload, compare with (Reichelt et al., 2009)). Similar protective effects are exerted by sparing reperfusion (Okamoto et al., 1986). From this point of view, the use of isoprenaline, which causes coronary vasodilation, seems more appropriate to us.

The study of I/R injury requires a specific approach to the adult Langendorff methodology, especially in the field of composition of the perfusion solution. Glucose levels, the amount of free or bound calcium, bicarbonate, pH and their mutual interactions should be as close as possible to the in vivo conditions, so that the results of cardioprotective interventions can be better defined.

The aim of this study was therefore to identify and characterize key methodological factors relevant for adult Langendorff experiments. Various concentrations of calcium (1.25 mM; 1.75 mM; 2.0 mM) and magnesium (1.2 mM; 0.6+ mM) were used to evaluate the response of isolated rat heart. pH and calcium activities, both in rat plasma and artificial solutions (including K‐H solution), were measured by ion selective electrodes (ISE). Spectrophotometric measurements were used to determine the normal range of total ions, albumin, total protein and phosphate in rats (protein binding affects ion activities). Osmolality of rat plasma was compared to that of the K‐H solution. Additionally, the effects of preload and catecholamine (isoprenaline) in Langendorff experiments were also described to determine whether they are necessary for these experiments.

## 2 METHODS

Due to the complexity of the experimental procedures, detailed methods are provided in Data S1. A concise summary is presented below.

### 2.1 Ethical approval

All animal experiments were conducted in accordance with the relevant guidelines and regulations and were approved by the Institutional Ethics Committee of 2nd Faculty of Medicine, Charles University, whose approval was a prerequisite for authorization by the Ministry of Education, Youth and Sports of the Czech Republic (MSMT). The final authorization for the experimental protocol was granted by the MSMT (decision no. MSMT‐15995/2023‐5). The Institutional Ethics Committee does not issue reference numbers.

### 2.2 Animals

Ninety adult male Wistar rats (300–480 g; corresponding to approximately 9–13 weeks of age based on standard growth curves) were used. Animals were housed under standard conditions with free access to food and water. To avoid hormonal influences on cardioprotection, only males were included. Anesthesia was avoided due to its potential effects on cardioprotection and calcium handling; euthanasia was performed by decapitation.

### 2.3 Langendorff heart preparation

Hearts were rapidly excised and perfused using a constant‐pressure Langendorff system (80 mmHg). A latex balloon (smaller balloons by the Radnoti or larger custom made) was inserted into the left ventricle to measure developed pressure (LVDP, LabChart, ADInstruments). The hearts were electrically paced at 330 bpm. After stabilization (20–25 min), global ischemia (40 or 80 min) was followed by 30 min reperfusion.

### 2.4 Assessment of ischemia–reperfusion injury

I/R injury was assessed by: (1) LVDP recovery, calculated as the percentage of pre‐ischemic values, and (2) TTC staining to quantify infarct size. Digital analysis of stained heart slices was performed by blinded researcher using 3D Slicer and Fiji ImageJ. Two color thresholds were used.

### 2.5 Computerized LVDP analysis

To evaluate for possible bias, LVDP data were also analyzed using a custom computational pipeline. This included signal filtering, artifact removal, and manual identification of pre‐ and post‐ischemic intervals for objective calculation of recovery (Matlab).

### 2.6 Preload and isoprenaline

Preload was applied using custom latex balloons. Isoprenaline (100 nM) was administered 5 min prior to ischemia and continued throughout reperfusion to evaluate its β‐adrenergic effects.

### 2.7 Perfusion solutions

The baseline Krebs–Henseleit solution contained (in mmol/L): NaCl 118, KCl 4.7, CaCl2 1.25, MgSO4 1.2, NaHCO3 20, KH2PO4 1.2, glucose 7, and mannitol 1.1. Solutions were oxygenated (95% O2/5% CO2) and maintained at 37°C throughout the experiment. Variants with modified calcium and magnesium levels were also tested (full details in Data S1).

### 2.8 Rat plasma and biochemical analysis

Plasma was obtained from blood collected post‐decapitation. Calcium, magnesium, albumin, protein, and phosphate levels were analyzed via spectrophotometry using commercial reagents (Biosystems). Ionized calcium activity was measured with a combined calcium‐selective electrode (Theta 90).

### 2.9 Artificial solutions and component testing

Simplified artificial solutions were used to evaluate the effect of individual components on calcium activity. Solutions were adjusted for ionic strength (osmolality). Dose–response assessments included control and two concentrations (low corresponding to the K‐H solution, high being triple that amount). Results are reported as mV (the obtained results challenge calibrations). Selected mV results are calculated to concentrations using regression from the K‐H and NaCl calibration curves.

### 2.10 pH and osmolality

Solution pH was measured with calibrated pH meters (Hanna, Theta 90). Osmolality of plasma and selected solutions was measured using a freezing‐point osmometer (Osmomat 3000 basic).

### 2.11 Statistical analysis

LVDP recovery and TTC results were evaluated using one‐way ANOVA and post hoc Student–Newman–Keuls test. Statistical tests were performed using R. Significance was defined as *p* < 0.05.

## 3 RESULTS

### 3.1 Baseline parameters

Body and heart weights and baseline contractile parameters are summarized in Table 1.

**TABLE 1:** Morphometry and cardiac function parameters of experimental animals; means ± SD.

Protocol	*N*	Body weight (g)	Heart weight (g)	HW/BW (mg/g)	LVDP (preisch, mmHg)
1.25	10	360.3 ± 17.52	1.976 ± 0.141	5.55 ± 0.42	57.61 ± 17.66
1.25p	10	355.8 ± 23.25	1.734 ± 0.101	4.88 ± 0.30	101.0 ± 11.70
1.75	10	359.6 ± 27.36	1.843 ± 0.195	5.02 ± 0.36	93.02 ± 22.52
1.75p	9	373.3 ± 25.94	1.892 ± 0.146	5.07 ± 0.25	125.5 ± 16.44
2.0	12	379.8 ± 21.30	1.906 ± 0.228	5.09 ± 0.48	71.99 ± 17.91
1.25 → 1.75	9	376.0 ± 49.55	2.039 ± 0.244	5.55 ± 0.32	—
Low Mg 1.75p	10	370.0 ± 13.81	1.706 ± 0.133	4.61 ± 0.35	98.46 ± 27.16
Isop. 1.75p	10	382.5 ± 14.40	1.873 ± 0.169	4.90 ± 0.44	136.7 ± 20.20
1.75p 80 min isch	10	367.4 ± 13.26	1.988 ± 0.119	5.42 ± 0.43	105.6 ± 13.10

### 3.2 pH of a Krebs–Henseleit solution

In agreement with Fantinelli and Gonzales et al. (Fantinelli et al., 2013; Gonzalez Arbelaez et al., 2016), K‐H solution with bicarbonate 25 mM is alkaline (even when saturated with CO_2_). Reduction of bicarbonate to 20 mM normalized the pH (7.4). Individual results are in the Table 2.

**TABLE 2:** pH Values of the K‐H solution depending on the bicarbonate (mM) and CO_2_ saturation, *N* = 1.

Bicarbonate	25 mM (14) without CO_2_	25 mM (14) with CO_2_	20 mM (13) without CO_2_	20 mM (13) with CO_2_
pH	7.94	7.68	7.87	7.40

*Note*: Parentheses () indicate solution number from the Table S1.

### 3.3 Relationship between TTC staining result and time of analysis

On increased length of ischemia group (after becoming suspicious of a possible issue here) we have determined that the extent of the decoloration is time dependent. Increasing the time the sample was submerged in the formalin solution to 7 and 10 days has significantly increased the white areas of the samples (Figure 2a,b, *p* < 0.001, *N* = 10).

### 3.4 Influence of the color threshold on the TTC analysis

Changing the color threshold by merely 15 points (on 0–255 axis) resulted in approximately double the “infarct size” (compare Figure 2a,b, *p* < 0.001 for each day).

### 3.5 Effect of calcium concentrations and preload on baseline LVDP and I/R injury

**FIGURE 1:**
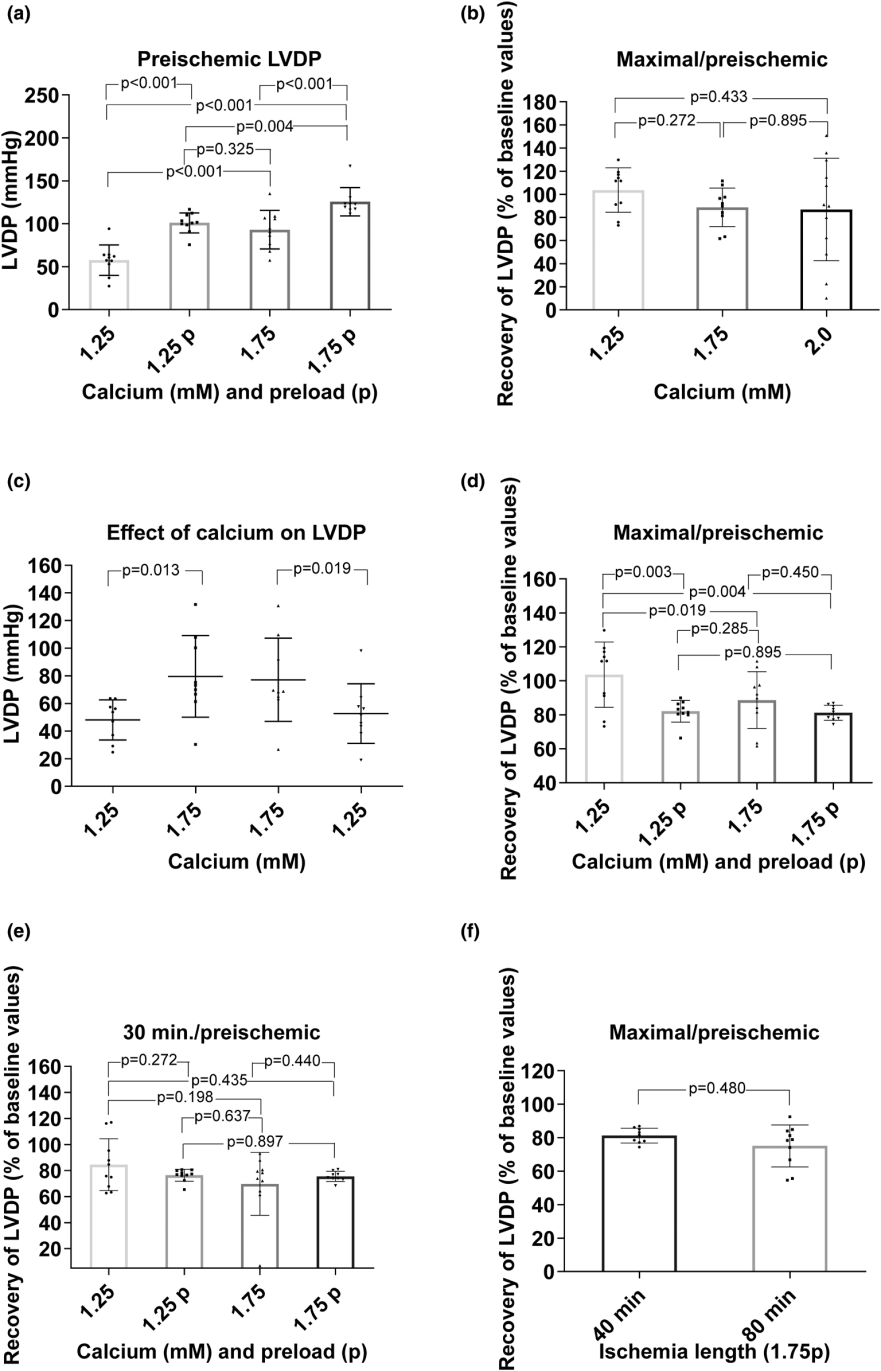
LVDP results of the Langendorff experiment. ANOVA with the Student‐Neumann‐Keuls test unless stated otherwise. (a) Dependence of preischemic LVDP on calcium levels and presence/absence of preload, *N* = 10,10,10,9 (b) Maximal LVDP recovery in various calcium concentrations, *N* = 10,10,12 (c) Effect of the change of the calcium concentration on the LVDP in the same heart, paired *t*‐tests, *N* = 9 (d) Maximal LVDP recovery with the addition of preload, *N* = 10,10,10,9 (e) 30 min. LVDP recovery with the addition of preload, *N* = 10,10,10,9 f) Maximal LVDP recovery in the prolonged ischemia, *t*‐test, *N* = 9,10.

**FIGURE 2:**
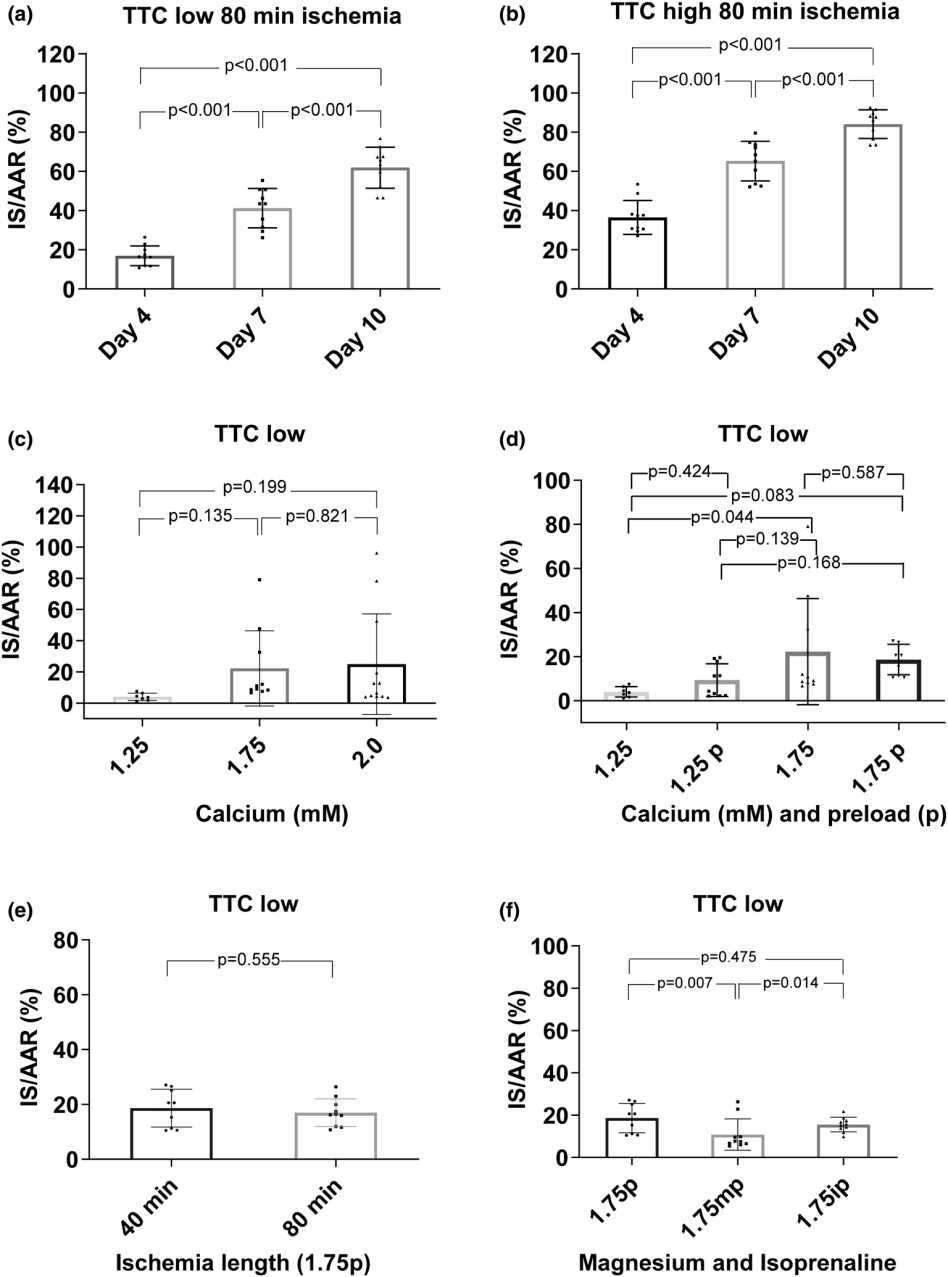
TTC results of the Langendorff experiments. ANOVA with the Student‐Neumann‐Keuls test unless stated otherwise. (a) Dependence of the TTC staining on sample storage time, stricter threshold, *N* = 10 (b) Dependence of the TTC staining on sample storage time, broader threshold, *N* = 10 (c) TTC staining in various calcium concentrations, stricter threshold, *N* = 7,10,12 (d) TTC staining with the addition of preload, stricter threshold, *N* = 7,10,10,9 (e) TTC staining (stricter threshold) in the prolonged ischemia, *t*‐test, *N* = 9,10 (f) TTC staining (stricter threshold) in the reduced magnesium and added isoprenaline groups, *N* = 9,10,10.

Calcium 1.75 mM was associated with significantly greater baseline LVDP values (compared to calcium 1.25 mM, Figure 1a, *p* < 0.001 *N* = 10,10). Results of I/R injury without preload did not reach statistical significance between calcium levels 1.25, 1.75, and 2.0 mM (Figures 1b and 2c, *p* > 0.05 on both LVDP and TTC).

In a different group we have also tested changing the calcium concentration on the same heart. Once again, calcium 1.75 mM was associated with the greater LVDP values compared to the calcium 1.25 mM (Figure 1c *p* = 0.013 and *p* = 0.019).

Addition of preload (p) to the experiments significantly increased I/R injury on low calcium levels (Figure 1d, *p* = 0.003). It also significantly reduced the variance of the results, creating significance in the results of previously non‐significant calcium groups (1.25–1.75 mM (Figure 1d, *p* = 0.019)). TTC staining confirms significant increase of the I/R injury in the calcium 1.75 mM compared to the calcium 1.25 mM (Figure 2d, *p* = 0.044).

### 3.6 Effect of ischemia length on I/R injury

**FIGURE 3:**
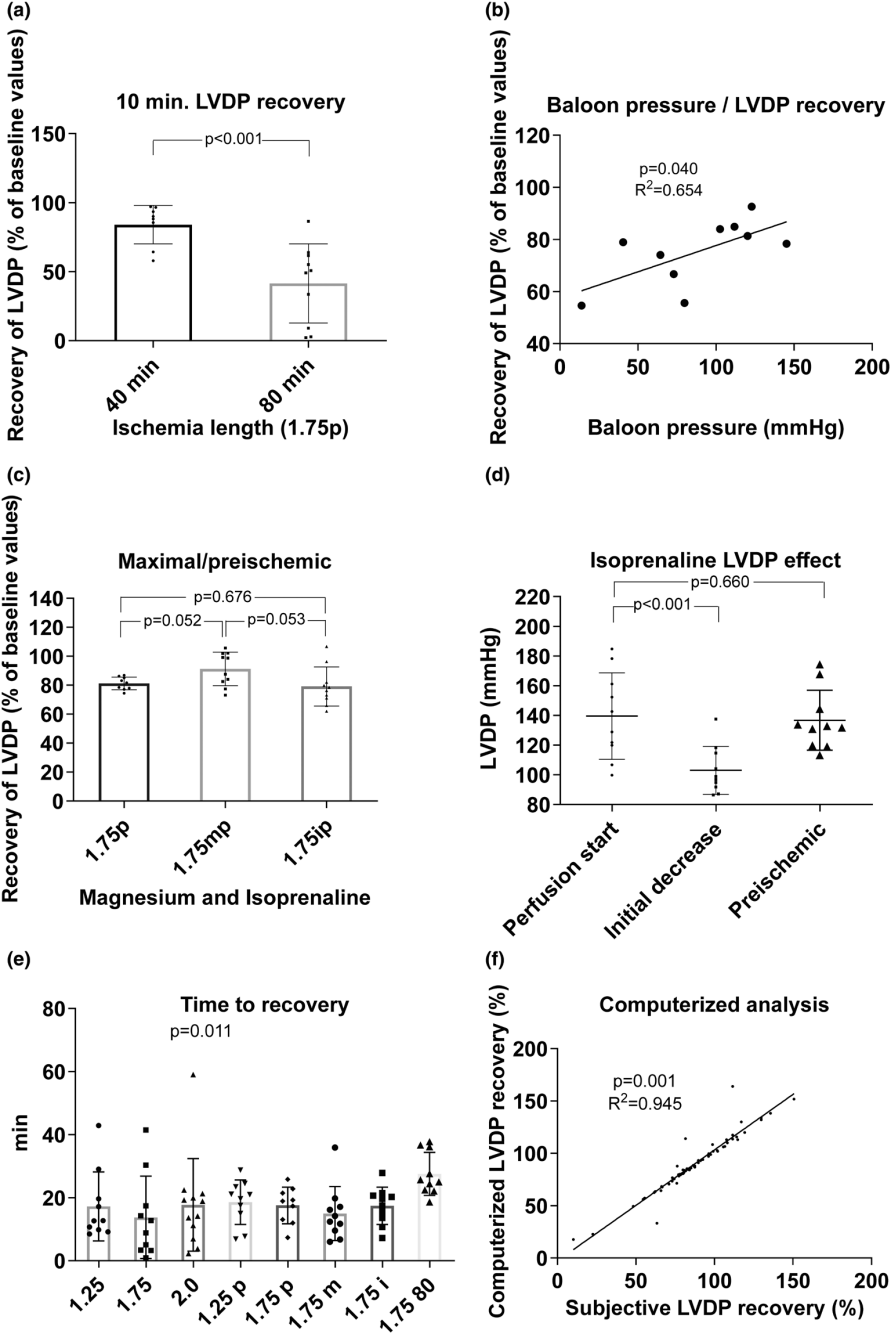
Additional results of the Langendorff experiments. ANOVA with the Student‐Neumann‐Keuls test unless stated otherwise. (a) 10 min. LVDP recovery in the prolonged ischemia, *t*‐test, *N* = 9,10 (b) Correlation between the balloon pressure and LVDP recovery, correlation with regression *N* = 10 (c) Maximal LVDP recovery in the reduced magnesium and added isoprenaline groups, *N* = 9,10,10 (d) Biphasic effect of the isoprenaline administration on the LVDP, *t*‐tests, *N* = 10 (e) Time to recovery (all groups), *N* = 10,10,12,10,9,10,10,10 (f) Computerized maximal LVDP recovery vs. subjective maximal LVDP recovery, correlation with regression, *N* = 81.

Increasing the ischemia length prolonged time to recovery (Figure 3e) so that the difference between “max” and “30 min” results became negligible. Notably, neither maximal nor “30 min” values of recovery were significantly reduced by the prolonged ischemia (Figure 1f, *p* > 0.05). TTC staining also yielded non‐significant results (Figure 2e, *p* > 0.05). There is a strongly significant difference in recoveries in “10 min” (*p* < 0.001), indicating greater I/R injury (probably including stunning and arrhythmias) compared to the lack of it in the 40‐min ischemia group (Figure 3a). Additionally, a significant correlation was found between balloon pressure and LVDP recovery values indicating that high balloon pressures limit I/R injury (Figure 3b, *p* = 0.040).

### 3.7 Effect of isoprenaline on LVDP and I/R injury

Consistent with the previous study on dobutamine (Ooiwa et al., 1989), isoprenaline did not increase I/R injury (Figure 3c and Figure 2f *p* > 0.05). Administration of isoprenaline caused immediate significant drop in the LVDP (*p* < 0.001), followed by an increase in the LVDP (Figure 3d). The overall effect of the isoprenaline on the LVDP was non‐significant (*p* > 0.05).

### 3.8 Rat total plasma calcium and magnesium

Rat total plasma calcium is >3 mM, and total magnesium is lower compared to the K‐H solution (~0.75 to ~0.9). Reduction of magnesium did not increase I/R injury (actually borderline reduced it, Figures 2f and 3c, *p* = 0.052 and 0.007 for LVDP and TTC respectively) and had significantly negative effect on the preischemic LVDP values (*p* = 0.012). The results of rat plasma biochemical values are in the Table 3.

**TABLE 3:** Results of rat plasma biochemistry and osmolality measurements.

Parameter/indicator/calibrator	Median	Q1	Q3	*N*	*n*
Calcium Cresolphtalein bovine (mM)	3.002	2.468	3.186	18	36
Calcium Cresolphtalein human (mM)	3.072	2.614	3.437	18	36
Calcium Arsenazo bovine (mM)	3.161	2.990	3.375	18	36
Magnesium Xilidyl blue bovine (mM)	0.8795	0.8045	0.9210	18	36
Magnesium Xilidyl blue human (mM)	0.7545	0.6813	0.8190	18	36
Albumin Bromocresol green bovine (g/L)	26.19	23.60	28.36	20	40
Albumin Bromocresol green water (g/L)	20.29	18.46	23.07	20	40
Total protein Biuret bovine (g/L)	59.93	56.25	65.88	20	40
Total protein Biuret water (g/L)	59.74	55.84	62.32	20	40
Phosphorus Phosphomolybdate bovine (mM)	1.638	1.371	1.878	20	40
Phosphorus Phosphomolybdate water (mM)	1.880	1.585	2.001	20	40
Osmolality (mOsm/kg)	299.5	295.3	301.0	10	10

*Note*: Median and interquartile range.

### 3.9 Rat plasma osmolality

Rat plasma shows higher osmolality (299.5 mOsm/kg, Table 3) compared to the K‐H solution (277 mOsm/kg).

### 3.10 Activity of calcium ions in the artificial solutions (excluding bicarbonate and phosphate)

**FIGURE 4:**
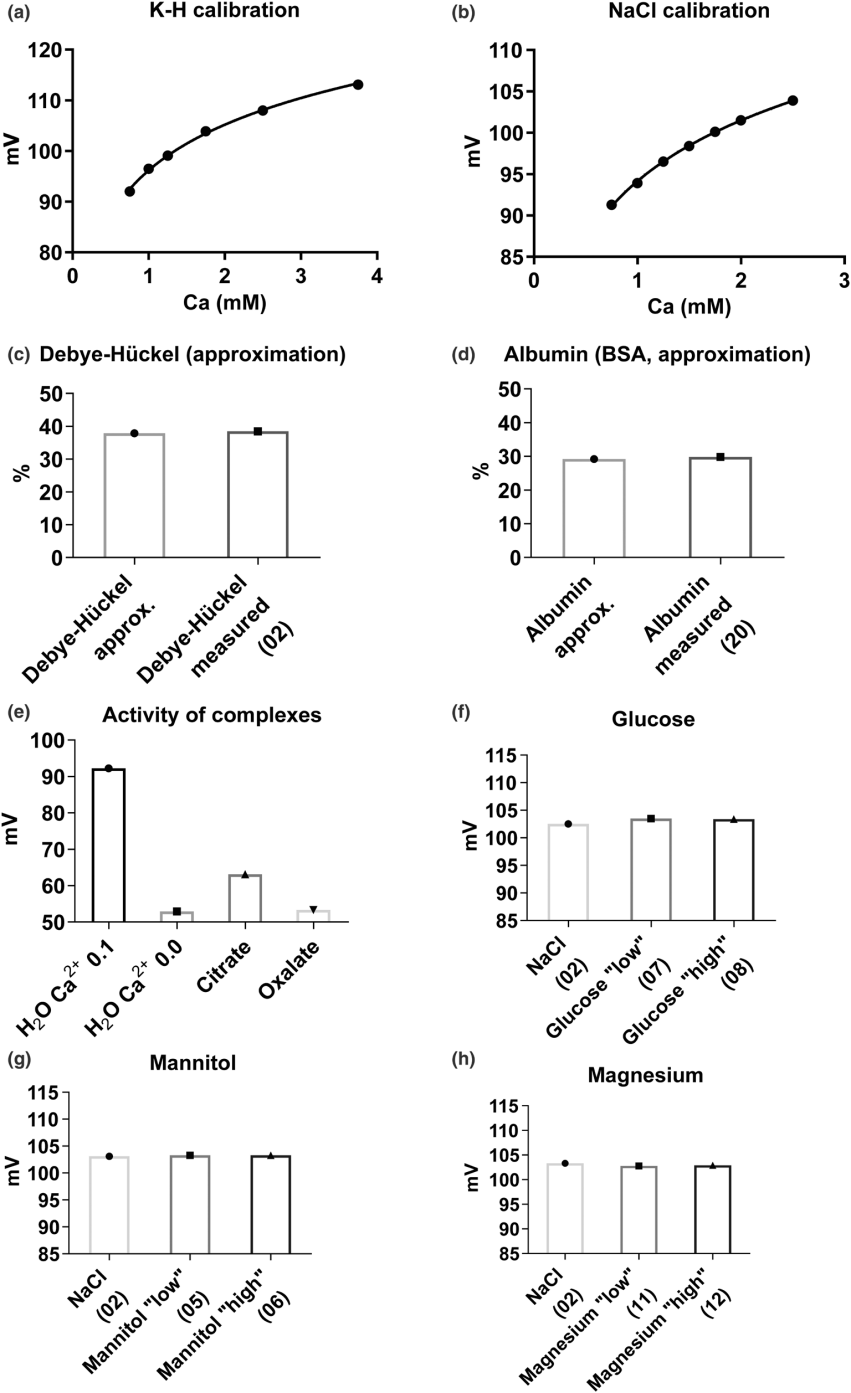
Calcium ISE measurements (parentheses () indicate solution number from the Table S1): *N* = 1 chemically defined artificial solution per experimental condition. These experiments were designed as physicochemical observations rather than biological replicate studies. (a) Krebs–Henseleit calibration curve (b) NaCl calibration curve (c) Approximation for the reduction in calcium activity caused by the Debye‐Hückel principle (d) Approximation for the reduction in calcium activity caused by the Albumin (e) Residual calcium activity in the distilled water saturated by citrate or oxalate (f) Influence of glucose on calcium activity (g) Influence of mannitol on calcium activity (h) Influence of magnesium on calcium activity.

The results of the Debye‐Hückel principle approximation and the addition of albumin (BSA) to solutions correspond to the expectations (Figure 4c,d). Calcium complexes with citrate or oxalate do not seem to be active (<<0.1 mM) (Figure 4e). Calcium activity was not affected by glucose, mannitol or magnesium (Figure 4f,g,h).

### 3.11 Influence of bicarbonate and phosphate on calcium activity in the artificial solutions

**FIGURE 5:**
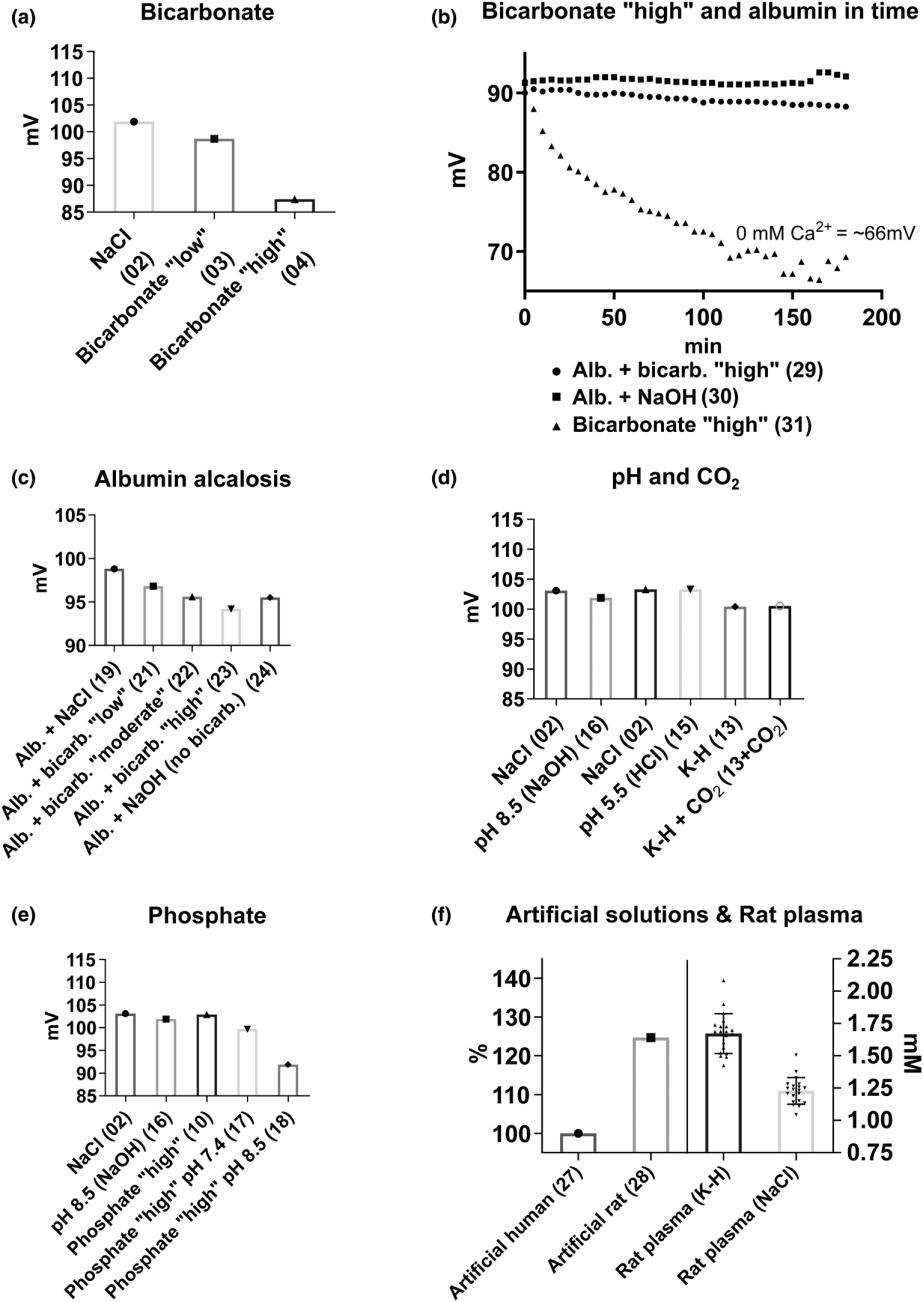
Calcium ISE measurements regarding bicarbonate (parentheses () indicate solution number from the Table S1) Unless otherwise indicated, *N* = 1 chemically defined artificial solution per experimental condition. These experiments were designed as physicochemical observations rather than biological replicate studies: (a) Dose‐dependent decrease in the calcium activity caused by bicarbonate (b) Time dependent decrease in the calcium activity in the “high” concentration of bicarbonate, albumin and bicarbonate with albumin (c) Comparison of the effects of alkalosis induced by bicarbonate or NaOH on calcium activity. (d) No effect on calcium activity of the isolated change of pH or CO_2_ (in the solution with nothing else to bind calcium to) (e) Influence of phosphate and pH on calcium activity (h) Comparison of calcium activity in the artificial solutions analogical to human and rat biochemistry (*N* = 1) and calcium activity in a rat plasma based on the Krebs–Henseleit and NaCl calibrations (*N* = 20).

Addition of bicarbonate causes a dose dependent drop in calcium activity (Figure 5a). Neither isolated change of pH (NaOH, HCl) nor CO_2_ saturation changes calcium activity (Figure 5d), likely indicating a direct formation of complexes of Ca^2+^ with bicarbonate. The amount of these complexes appears to be >20% (21.86% calculated from the K‐H calibration; 26.09% calculated from the NaCl calibration). This exceeds the expectations from the physiology of plasma (total complexes = <10% (Buckley & Russell, 1988)). Bicarbonate in the high concentration is not stable at the 15‐min result and continues to further reduce the calcium activity until values of ~0 mM are reached after several hours (Figure 5b).

*Dihydrogen* phosphate does not reduce the calcium activity. After being buffered, monohydrogen phosphate is created, which does reduce the calcium activity (in a pH dependent manner) (Figure 5e). However, the results of phosphate in the “high” concentration approximately correspond to the results of bicarbonate in the “low” concentration (3.4 vs. 3.2 mV), suggesting that bicarbonate is the most relevant substance in the actual experimental settings.

### 3.12 Relationship between albumin (BSA) and bicarbonate activity

Alkalosis in solutions containing albumin (BSA in our case) leads to a dose dependent drop in calcium activity. In solutions with albumin, comparing alkalosis caused by bicarbonate to alkalosis caused by NaOH yields greatly diminished differences (~1.3 mV, Figure 5c). This contrasts sharply to the results of bicarbonate in the absence of albumin (14.5 mV, Figure 5a, indicating an approximately 58–66% drop in concentration based on the calibration curve used) which strongly suggests powerful bicarbonate‐albumin interaction, limiting the activity of bicarbonate.

The solution of albumin with high bicarbonate is still not stable in time and continues to decrease calcium activity over time; however, the magnitude of that drop is greatly diminished (2.1 mV vs. 23.9 mV in ~3 h). This is in contrast to albumin with NaOH (at the same pH), which is stable over time (0 mV in ~3 h, Figure 5b).

Hypoalbuminemia is clinically believed to cause alkalosis (McAuliffe et al., 1986). However, when pH is shifted to acidic (HCl addition) and albumin is removed, hypoalbuminemia aggravates acidosis (Table 4). This suggests that the primary role of albumin is buffering of any changes already present in the solution, rather than acidification/alkalization directly.

**TABLE 4:** pH values of solutions containing albumin.

Solution	A 40 B 0 (19)	A 40 B 25 (32)	A 20 B 25 (33)	A 0 B 25 (34)	A 40 pH 6.5 (25)	A 0 same HCl (26)
pH	6.88	7.46	7.62	8.25	6.59	4.38

*Note*: Parentheses () indicate solution number from the Table S1.

Abbreviations: A, albumin (g/L), B, bicarbonate (nM), pH, HCl titration.

### 3.13 Rat albumin, total protein, and phosphorus levels

Rat plasma contains lower albumin (20–27 g/L), borderline low total protein (~60 g/L) and higher phosphate concentrations (~1.6–1.8 mM) compared to human plasma (Table 3). Results of albumin (bromocresol green) show discrepancy between serum and water calibrations, with control serums speaking in favor of the water calibration results (i.e. ~20 g/L).

### 3.14 Activity of calcium in rat plasma and estimated comparison to humans (in the artificial solutions)

The artificial solution (see Table S1) analogical to the measured rat plasma biochemistry parameters shows approx. 24.7% higher Ca^2+^ activity compared to the artificial solution analogical to the human plasma biochemistry (Figure 5f). Calcium activity in rat plasma is 1.686 mM based on the K‐H calibration and 1.236 mM based on the NaCl calibration (Figure 5f).

## 4 DISCUSSION

The main result of our study is the explanation of the non‐physiological chemical behavior of the Krebs–Henseleit solution. We have observed much higher capacity of bicarbonate to bind calcium than expected (>20%) with reduced calcium activity affecting experiments performed using the K‐H solution. Moreover, this capacity was severely diminished in the presence of albumin. This phenomenon was present without any change in bicarbonate concentration. In the past, several other theories have been proposed to explain the ABB behavior of albumin (Stewart‐Fencl, substitution of albumin by bicarbonate etc. (Fencl et al., 2000)), but our findings raise the possibility that it is actually the buffering capability of albumin (Giosa et al., 2023), rather than direct acidification or alkalization that plays the primary role. A lack of albumin leads to a substantial increase in bicarbonate activity, even without any change in its concentration. While the precise nature of the albumin‐bicarbonate interaction is not entirely clear to us, it is known that albumin can bind other negatively charged molecules, such as salicylate (Parsons & Sathe, 1991). The other observed calcium activities in the artificial solutions align with expected behavior reported in previous studies (Hu et al., 2018). Thus, while not directly tested here, our findings raise the possibility that albumin may buffer bicarbonate activity more significantly than previously appreciated.

This difference is partly explained by the higher physiological calcium activity in rat plasma compared to human plasma. Our measured values of total calcium in rats are consistent with previously reported data (Boguszewska‐Czubara et al., 2011) and with the reference values for Wistar rats provided by Charles River Laboratories (12.67 mg/dL ~3.16 mmol/L). High total calcium and low total albumin participate on the higher ionized calcium. This effect also appears to be approximately ≥20%. Combination of the higher ionized calcium in rat plasma and more bicarbonate complexes in the K‐H solution therefore indicates roughly ≥40% difference, which is strikingly similar to the actual difference between calcium levels of 1.25 and 1.75 mM. The measured calcium activity in rat plasma further rules out Ca^2+^ 1.25 mM in the K‐H solution as physiological. Although the value is slightly lower than 1.75 mM, we emphasize that some results of the plasma collection from decapitations likely were hemolytic, and the accuracy of the pH correction cannot be fully confirmed and minor deviations cannot be excluded. The only result potentially counteracting the higher calcium activity would be the higher phosphate in rat plasma. While we have not performed any tests regarding a “calcium‐phosphorus product”, this concept has already been criticized by the nephrological studies (e.g. ectopic calcifications do not correlate with it (O'Neill, 2007)). In summary, these data explain the behavior of rat heart in the Langendorff apparatus, where the concentration of Ca^2+^ of 1.75 mM was associated with greater LVDP values and I/R injury in agreement with observations by Reichelt (Reichelt et al., 2009). Our results therefore indicate the necessity to appropriately adjust calcium concentrations in the artificial solutions. We also recommend preparing and using K‐H solution on the same day (the difference in one day old solution appears to be ~1 mV).

Our measurements indicate low levels of rat plasma albumin (and protein) compared to the humans. Other studies have reported rat plasma albumin ~30 g/L (Natali et al., 2000; Sethi et al., 2019), which is somewhat similar to our findings of 27–25 g/L in the serum calibration (calibration was not described in those studies). Bromocresol green is known to overestimate actual results and immunological measurement would most likely be needed to verify these findings (van Schrojenstein Lantman et al., 2023) (dye also reacts with globulins to a lesser extent (Filípek & Illek, 2020)). We have considered other explanations (starvation), but the rats were gaining weight normally and nothing out of ordinary was reported by our menagerie. Moreover, a study analyzing hypoproteinemic starvation in rats concluded that it leads to the drop of the globulins while albumin levels remain intact (Natali et al., 2000). Our results were also consistent in two groups of rats and with two different bottles of calibration/control serums. The discrepancy between water and serum calibrations should be further evaluated in future Bromocresol green measurements; however, the underlying cause of this discrepancy remains to be clarified and warrants further investigation, potentially involving quantitative assays for albumin measurement, such as ELISA or immunoturbidimetric methods.

In our study, we found that the pH of the Krebs–Henseleit (K‐H) solution remained alkaline despite containing a bicarbonate concentration (25 mM) considered physiological and being equilibrated with 5% CO_2_. This effect was observed even though no change in total bicarbonate concentration occurred during solution handling, indicating that factors other than concentration may influence the buffering environment of the solution. A similar phenomenon has been described in clinical and experimental studies of hypoalbuminemia, where alkalosis also develops (Park & Sidebotham, 2023; Rossing et al., 1986). However, while the classical Henderson–Hasselbalch equation (effectively a simplification of Guldberg and Waage's Law of Mass Action) remains widely used to predict pH, its underlying assumptions, namely, that “concentrations approximate activities” and that “activity of a substance is not affected by the presence of other substances in the solution”, may not hold in hypoproteinemic or protein‐free solutions such as K‐H buffer. Moreover, bicarbonate concentration is typically a *calculated* parameter (not directly measured), and calculation by the Henderson–Hasselbalch equation cannot distinguish increased concentration from increased activity. Calculations based on the electroneutrality principle (AG) may be biased by this problem, and this was noted before (Fencl et al., 2000). We hypothesize that the absence of protein‐mediated buffering in the K‐H solution results in an increased apparent activity of bicarbonate ions, leading to higher pH than would be expected under physiological conditions. This could reconcile the discrepancy between the need to reduce bicarbonate in K‐H solutions to avoid alkalinity, and the observation that rat plasma normally contains even higher bicarbonate levels without inducing alkalosis. Notably, recent studies have independently opted to reduce bicarbonate concentration in perfusate to ~20 mM, likely in response to similar considerations (Al‐Othman et al., 2024). In conclusion, adjusting bicarbonate levels downward in artificial perfusates may improve physiological relevance in Langendorff‐based I/R studies.

It should also be noted that the “high” bicarbonate solution with albumin was not stable over time either, although the scale of the drop was greatly diminished by the presence of albumin. In contrast, the NaOH alkaline solution with albumin remained stable over time. The majority of our results do not take time as a factor and need to be viewed as such. Time is probably not a factor when considering binding of calcium to albumin, but calcium activity continues to drop in the bicarbonate solution even with albumin, albeit at a much slower rate. This effect could be further reduced by the presence of additional buffers (such as globulins), which we cannot account for. We are therefore not making any conclusions regarding ionized calcium in alkalosis in the actual clinical settings as to what extent albumin, other proteins, and bicarbonate play a role.

Our results of osmolality measurements are consistent with the historical measurements (Dunn et al., 1973). However, since obtaining these results required to specimens, we did not correct this parameter in this study. We are considering using modified K‐H solution with increased mannitol to correct for this parameter in the future. However, we are also concerned that this might be detrimental to cardioprotection (hyperosmotic environment is known to negatively impact cardioprotection (Zalesak et al., 2016)). In contrast, magnesium is high in the K‐H solution relative to the rat plasma and from the in vivo studies we were highly suspicious that this might also affect I/R injury (Matsusaka et al., 2002; Ravn et al., 1999), however testing for that parameter in the Langendorff has yielded negative results. This indicates that not all parameters necessarily affect I/R injury, even if they are incorrect.

Regarding the Langendorff methodology, the first key finding is the preload, which increased I/R injury at low calcium levels but did not have any effect on I/R injury at correct calcium levels. However, it also significantly reduced the variability of the results. We were originally hoping that our data would also help to clarify the conflict between “energy consumption,” “calcium overload” and I/R injury, where it remains uncertain whether the calcium overload is a result of energy depletion or an independent and possibly active mechanism (Shintani‐Ishida et al., 2012). Isoprenaline did not increase I/R injury either, but calcium did. However, since calcium also increased the force of contraction (LVDP) and cAMP pathway is known to be linked to calcium (Schmid et al., 2014; Taylor, 2017), we are still not sure about the interpretation. Additionally, the 30‐min results overall show higher values of preload groups compared to non‐preload groups, although this difference is not significant probably due to the insufficient sample size to prove interaction (*p* = 0.085) (Figure 1e vs. Figure 1d). This is probably due to the absence of preload causing drop in LVDP in the 30 min, which is not actually caused by the I/R injury. Overall, we suggest preload should be used as a precaution and also to reduce inaccuracies in the LVDP results and to maintain LVPD more stable during the reperfusion.

Surprisingly, isoprenaline administration leads to a lack of LVDP increase. This result is, however, also consistent with the previously obtained data. A lack of LVDP increase and a reduction of coronary flow after isoprenaline administration (in paced hearts) were observed in this study (Ozakca et al., 2013). These findings highlight a known limitation of the isolated ex vivo preparation, in which β‐adrenergic responsiveness may differ substantially from the intact in vivo heart. Differences in oxygen delivery and coronary regulation in crystalloid‐perfused and sympathetic‐denervated hearts may contribute to these observations. The underlying mechanisms were not addressed in the present study.

Additional concern appears to be the length of the ischemia, where results after 40 min ischemia showed rapid recovery of the LVDP, followed by an actual drop to 30 min (a common time point for reporting “results”). However this pattern changed with increasing the length of ischemia showing marked decrease of LVDP during early reperfusion (consistent with expected stunning and/or arrhythmias) followed by the recovery of LVDP. We should also point out that in the actual clinical settings of myocardial infarction, the “symptoms onset to needle time” was reported to be 3.2 h (Hromadka et al., 2022). Additionally a recent review mentions that the I/R injury remains reversible during the initial 20–40 min and irreversible damage (e.g. necrosis) develops when the coronary occlusion is longer than 20–40 min (Heusch, 2024). While metabolic scaling is often cited to justify shorter lengths of ischema in rodents, there is a notable lack of systematic experimental data evaluating longer no‐flow ischemia in adult Langendorff preparations. Therefore, in our opinion, these findings suggest that ischemic lengths in most Langendorff studies are insufficient.

Our results suggest that TTC quantification may be sensitive to methodological details such as fixation time and thresholding. Longer fixation time lead to greater sample discoloration and small changes in thresholding caused significant changes in the reported result. Other studies were reporting ~50% infarct size while using 40 min of global ischemia (Babiker et al., 2019). These discrepancies between functional and histological outcomes underscore the need for further validation of TTC‐based infarct quantification protocols in global ischemia models. We propose standardized protocols and further validation studies to enhance its reliability. Comparing our results with the LVDP, the stricter threshold appears to be more accurate to us.

We have also evaluated the time to recovery (in the “max” LVDP measurements) and contracture development in our experiments. The correlation between the time to recovery and actual LVDP recovery value is significant, yet very weak (*p* = 0.0005, *R* ^2^ = 0.1640). Time to recovery did not detect any differences (Figure 3e). In the 80‐min ischemia group, the time to recovery was very close to 30 min (27:32), erasing differences between “max” and “30 min” recoveries. The contracture amplitude yielded significant ANOVA with non‐significant Newman–Keuls test. Time to onset of ischemic contracture has been criticized in the past (Ostadalova et al., 1998). We are therefore of the view that these methods are not suitable to determine I/R injury. We have also compared the computerized analysis to the unblinded analysis, yielding negligible differences (Figure 3f). The LVDP analysis was not blinded simply because of lack of personnel to do so (blinding the TTC analysis was prioritized, since a major issue there was expected).

There are some limitations to our study. While we uncovered methodological issues with the TTC staining, our study still used a time interval, so our results could still be partly affected. We cannot determine with certainty which time or color threshold is appropriate for the analysis. In the future, we propose even stricter criteria (e.g. single time‐point to take the results). Additionally, our 1.7p 80 min group results are likely also artificially high, due to the high pressures required to inflate balloons that were too small. Our data suggest that standard balloon sizes offered by the Radnoti may not provide optimal preload in our setup. Correlation between balloon pressure and LVDP recovery shows statistically significant pattern with recovery increasing as pressure increases. Gentle reperfusion alone is known to have protective effect (Okamoto et al., 1986). Furthermore, while our results suggest the possible albumin‐bicarbonate interaction, we are not equipped to study such chemical phenomenon further nor can we make any conclusions regarding hypoalbuminemic alkalosis in the clinical settings. Finally, accurate quantification of pH is constrained by the intrinsic properties of glass ion‐selective electrodes. These electrodes respond to hydrogen‐ion activity rather than concentration. Variability can arise from liquid‐junction potentials, aging of the hydrated glass layer, minor reference‐electrode drift and possibly slow CO_2_ equilibration. These factors may have contributed to the inconsistencies observed in our own and previous measurements of absolute pH in Krebs–Henseleit solutions. For this reason, the present study reports relative changes (ΔpH, Table 4) between solutions rather than relying on absolute values that could be affected by electrode‐ and matrix‐dependent artifacts. We also acknowledge that Langendorff preparations do not recapitulate the full in vivo environment (e.g. inflammatory components, red blood cells), but allow controlled testing of specific mechanistic hypotheses.

The primary purpose of this study was, however, to address the issue of the failed translation of preclinical results to clinical medicine. Cardioprotective interventions were tried in the clinical medicine in the past with mixed results (Dwyer et al., 2013; Freixa et al., 2012; Piot et al., 2008; Staat et al., 2005; Stiermaier et al., 2019), however major (i.e. multicentric) studies have yielded negative results (Cung et al., 2015; Hausenloy et al., 2019; Ottani et al., 2016). We are of the view, that this is caused by the discrepancies summarized in Table 5, which however can be corrected in the future studies. Apart from the already mentioned factors (calcium, preload, length of ischemia) we want to briefly discuss sex and comorbidities. Preclinical studies often insist on using both sexes. However, actual female patients in clinical studies are postmenopausal, unestrogenized and with comorbidities. This in our opinion creates a situation where data from premenopausal females cannot be applied even in postmenopausal females (refer to this study (Babiker et al., 2019), Figure 3, showing a failure of female sex to be protective in the presence of a severe comorbidity). Furthermore, various comorbidities negatively interfere with cardioprotection, including hypertension (Gonzalez Arbelaez et al., 2016; Wagner et al., 2013), dyslipidemia (Giricz et al., 2009), obesity (Wensley et al., 2013), diabetes (Wagner et al., 2008) (and hyperglycemia alone (Torregroza et al., 2021)), heart failure (Ma et al., 2013) and liver cirrhosis (Moheimani et al., 2021). Metabolic factors also appear to play important role in the cardioprotection (Louzada et al., 2021). Particularly SHR rat strain is of interest to us, previously it was shown to be fully resistant to cardioprotection in vivo (Wagner et al., 2013), but only partially resistant to cardioprotection in the Langendorff with calcium 1.35 mM (Gonzalez Arbelaez et al., 2016). A different study in the Langendorff with calcium 1.5 mM yielded not significant IPoC results (Penna et al., 2010). Since we want to study comorbidities further, we have concluded, that using the incorrect environment in the Langendorff (such as low calcium) would likely lead to a failure of such study. Therefore, clinical studies should be mindful of these discrepancies and address them (combine protective interventions, sufficient acute treatment of comorbidity (Calvert et al., 2008), active search for a bimodal distribution and confounders in the results) rather than find p slightly higher than 0.05 and report results as “not significant” (Dwyer et al., 2013). If an initial treatment of a hypertension yields unsatisfactory results, additional drugs are added to create a combination treatment.

**TABLE 5:** Comparison of conditions potentially affecting cardioprotection with clinical medicine.

	Preclinical studies	Clinical studies
Calcium	Low	Normal
Preload	Absent	Present
Ischemia length	20–40 min	3+ hours
Females	Premenopausal, estrogenized	Postmenopausal, unestrogenized
Comorbidities	Usually absent	Present

Taken together, our findings indicate that even moderate alterations in the composition of perfusion solutions, particularly in bicarbonate and albumin content, can substantially influence buffering capacity and ion activities. This observation may explain some of the inconsistencies in Langendorff protocols reported in the literature (Reichelt et al., 2009) and emphasizes the importance of methodological harmonization. By elucidating these biochemical effects, our work complements previous studies and offers a basis for optimizing perfusion conditions in experimental cardiac models. Further research should explore whether adjusting preload and length of ischemia can enhance the translational relevance of such models to clinical settings.

## 5 CONCLUSION

Our results highlight that even moderate changes in the composition of perfusion solutions, particularly in bicarbonate and albumin content, can markedly influence their buffering capacity and resulting ion activities. This may account for inconsistencies in reported protocols and underscores the need for methodological standardization. By clarifying these biochemical effects, our study provides a practical framework for optimizing perfusion conditions in experimental cardiac models. Additionally, optimization of use of preload and length of ischemia may further improve the relevance of experimental settings in terms of possible transfer to clinical medicine.

## AUTHOR CONTRIBUTIONS

**Jan Doul:** Conceptualization; data curation; formal analysis; investigation; methodology. **Zuzana Charvátová:** Data curation; formal analysis. **Martin Modrák:** Formal analysis; methodology. **David Kala:** Funding acquisition; methodology; software. **Jakub Otáhal:** Conceptualization; funding acquisition; methodology; supervision.

## FUNDING INFORMATION

Support of this work was provided through project no. LX22NPO5107 from the MEYS of the Czech Republic: Financed by EU—Next Generation EU.

## CONFLICT OF INTEREST STATEMENT

The authors have no relevant financial or non‐financial interests to disclose.

## ETHICS STATEMENT

All investigations conformed to the “Guide for the Care and the Use of Laboratory Animals”, published by the US National Institutes of Health. Both the Animal Studies Committee of the Second Faculty of Medicine, Charles University and the Ministry of Education, Youth and Sports of the Czech Republic approved this study (decision number MSMT‐15995/2023–5). This article does not contain any studies with human participants performed by any of the authors.

## Supporting information


Data S1.
**Table S1.** Composition of the solutions for the ISE measurements (mM unless stated otherwise), (*t*) = titration grams are per 100 mL.

## Data Availability

The dataset supporting the conclusions of this article is available in the Figshare repository [DOI:10.6084/m9.figshare.28368329].
