# Supplementary material for: Impact of bicarbonate on ionized calcium and cardiac function in albumin‐free Langendorff perfusion in hearts of male rats
Source: Physiol Rep. 2026 Aug 10;14(15):e71053. doi: 10.14814/phy2.71053 (PMC13457654; doi:10.14814/phy2.71053)
Supplement: Supplementary file 1 [file PHY2-14-e71053-s001.docx]

**Impact of Bicarbonate on Ionized Calcium and Cardiac Function in Albumin-Free Langendorff Perfusion in Hearts of Male Rats.**

*Experimental Physiology*

**Jan Doul^1^*, Zuzana Charvátová^1^, Martin Modrák^2^, David Kala^1^, Jakub Otáhal^1^**

^1^Department of Pathophysiology, Second Faculty of Medicine, Charles University, Prague, Czechia

^2^Department of Bioinformatics, Second Faculty of Medicine, Charles University, Prague, Czechia

***Corresponding author:**

Jan Doul

jan.doul@lfmotol.cuni.cz

Department of Pathophysiology

Second Faculty of Medicine, Charles University

Plzeňská 130/221 150 06 Praha 5

Czech Republic

Phone: +420 257 296 410

**ORCID:**

Jan Doul: 0000-0003-0901-2270 jan.doul@lfmotol.cuni.cz

Zuzana Charvátová: 0009-0006-4806-3379 zuzana.charvatova@lfmotol.cuni.cz

Martin Modrák: 0000-0002-8886-7797 martin.modrak@lfmotol.cuni.cz

David Kala: 0000-0002-3010-1174 david.kala@lfmotol.cuni.cz

Jakub Otáhal: 0000-0001-5944-5803 jakub.otahal@lfmotol.cuni.cz

**Detailed methods**

*Ethical approval*

All animal experiments were conducted in accordance with the relevant guidelines and regulations and were approved by the Institutional Ethics Committee of 2^nd^ Faculty of Medicine, Charles University, whose approval was a prerequisite for authorization by the Ministry of Education, Youth and Sports of the Czech Republic (MSMT). The final authorization for the experimental protocol was granted by the MSMT (decision no. MSMT-15995/2023-5). The Institutional Ethics Committee does not issue reference numbers.

*Animals*

A total of 90 adult Wistar rats (Velaz) were used throughout the experiments. Animals were ordered by body weight rather than age and housed for 1–3 weeks prior to experimentation. Based on standard growth curves for male Wistar rats, the body weight range used (300-480 g) corresponds to an estimated age of approximately 9–13 weeks at the time of experiments. Two animals with higher body weight (440 g and 478 g) were housed longer than the remaining cohort and represented exceptions within the 1.25→1.75 mM calcium group - these animals were not compared with other experimental groups. All animals had free access to water and a standard laboratory diet (Altromin 1324, Velaz) ad libitum. Animals were housed in standard housing conditions on a 12-hour light/12-hour dark cycle. In order to avoid influencing the cardioprotection with estrogens, only males were used, and the female sex was excluded from this study. Since many anesthetics influence the cardioprotection or calcium metabolism, we have decided to use decapitation instead.

*Langendorff perfused heart*

The animals were weighed, then killed by decapitation. The chest was quickly opened and an epoxy-coated stainless steel cannula (14G; 16G if cannulation with 14G proved difficult) was inserted into the ascending aorta and perfused with normal saline. The heart was then rapidly excised and perfused in the Langendorff mode under constant pressure of 80 mmHg. The atria were removed and a catheter with latex balloon was inserted into left ventricle. Baloons supplied by Radnoti (Langendorff manufacturer) for rat are too small to give any preload to the heart (3 x 7 mm, non-expandable) and therefore just measure the pressures inside left ventricle. Left ventricle developed pressure (LVDP) was determined by calculating the difference between systolic and diastolic pressures.

The hearts were electrically paced at a rate of 330 beats/min (approximately the physiological heart rate (Azar et al., 2012)) using steel electrodes attached to the base of the heart. The stimulation was performed with pulses of alternating polarity, 2 ms duration and 3 mA current. The pressure inside the balloon was gradually increased until maximum LVDP values were reached. LVDP was continuously measured using a pressure transducer (MLT0380/D, ADInstruments) connected to the Power Lab and the computer (LabChart 7 software, ADInstruments). Following a stabilization period (20-25 min) the hearts were subjected to 40 or 80 min of ischemia followed by 30 min of reperfusion.

*Assessment of Ischemia-Reperfusion Injury*

We used two methods to evaluate I/R injury — the recovery of LVDP pressure and TTC staining. The recovery of LVDP pressure was calculated as a percentage difference between recovery values (calculated as maximum recovery and as 30 min recovery) and preischemic values (the value right before the start of the ischemia). These recovery values were then compared to the results of TTC staining to determine which method more accurately reflects the extent of I/R injury. At the end of the Langendorff experiment, the hearts were weighted.

The ventricles were then sliced into 2 mm sections and incubated for 2 10-minute periods in 2% TTC solution before being placed into 10% formalin (4% formaldehyde) solutions for 2-4 days (unless stated otherwise). Both sides of the slices were then photographed using stereomicroscope with a digital camera and photographs were further analyzed by computer using 3D Slicer and Fiji Image J software. In 3D Slicer, the outer boundary of each slice was outlined, turning anything outside the heart black. The color thresholding in Fiji Image J was then used to determine necrotic (white) and viable (red) areas. The “Lab” color space was selected to determine the ischemic areas, because it distinguishes normal and ischemic areas better than other methods. Two different thresholds (155 = “fully white”, marked as “*low*” in the results; and 170 = “slightly white”, marked as “*high*” in the results) were used for the analysis, because we are concerned, that counting “slightly white” areas as fully ischemic overestimates the actual infarct size. The researcher performing this analysis was blinded (each threshold was selected subjectively by the researcher, but the same value was used for each section).

*Computerized analysis of LVDP*

To evaluate the possible subjective bias of LVDP analysis (unblinded) a computerized analysis was developed for comparison. Variance of the LVDP channel was obtained in Labchart and calculated channel is obtained by nullifying parts of LVDP curve with increased variance (“Threshold(Smoothsec(Window(ch10;-1;1);0,3);1)*ch8“, channel 8 being the calculated LVDP). This eliminates artifacts from the recording. This adjusted curve is then exported into Matlab (R2021b). Maximum values are then obtained simply as “max” function in the selected part of recording. Pre- and post-ischemic values were semi-automatically detected from the raw data curve by following pipeline. The raw data, sampled at 1 kHz, were initially processed using a 1D median filter with an order of 10,000 to reduce noise. Outliers were subsequently removed using a Hampel filter of the same order, and missing values were replaced using B-spline interpolation. The filtered data were further smoothed with a moving average filter of the same order to retain only the primary features of the signal. The Douglas-Peucker algorithm, with a tolerance of 10, was then applied to the filtered curve to extract key points representing significant changes in the curve trajectory. From these key points, those corresponding to the pre-ischemic and post-ischemic phases were manually selected. 30-minute value is then calculated by adding 1800000 to the post-ischemic value, (30 minute value equals 30 min * 60 (sec. per minute) * 1000 samples per second). If the 30-minute value ends up being 0 (because there was artifact that was deleted as described previously), researcher has to manually search for the closest value that is not deleted. Apart from that (and the fact, that pre-ischemic and post-ischemic area is selected by the researcher, but this of course cannot create any bias), this analysis produces final results without any option for any subjective bias by the analyzing researcher.

*Preload and isoprenaline*

To obtain results with preload, custom-made latex balloons of a larger size (~5 x 7 mm, condom tip, expandable) were made. They were inserted into ventricles as described previously and gradually inflated until maximum LVDP values were obtained (further increase in pressure inside balloon leads to drop of LVDP). This procedure aligns with the Radnoti guidelines regarding adult Langendorff and differs from our previous neonatal studies, where preset levels of preload were used.

Isoprenaline was used to obtain β2 coronary dilation alongside β1 agonist effects. A concentration (100 nM) was selected according to the previous study (Schreckenberg et al., 2009). Perfusion started 5 minutes before ischemia and continued during entire reperfusion (ensuring that both preischemic and reperfusion LVDP values were influenced, allowing for a comparison of their ratio).

*Variations of the Krebs-Henseleit solution*

This study includes several variations of the Krebs-Henseleit solution. The base version was chosen according to our neonatal studies and contained (in mmol/l): NaCl 118.0; KCl 4.7; CaCl_2_ 1.25; MgSO_4_ 1.2; NaHCO_3_ 20.0; KH_2_PO_4_ 1.2; glucose 7.0 and mannitol 1.1 (Doul et al., 2024). All solutions were saturated by a mixture of 95% O_2_ and 5% CO_2_ (pH 7.4) in the reservoir and was further reoxygenated by the sheet flow oxygenating chambers at the top of the apparatus. Temperature was maintained at 37 °C (tubes were water jacketed). The perfusate was not recirculated.

The only difference from our previous studies was the reduction of NaHCO_3_ from 25 to 20 mM. This change was made based on our pH measurements, which indicated alkaline nature of the saturated K-H solution. This adjustment was implemented prior to all the reported experiments and was consistently maintained throughout the study. Further variations of K-H solution include increasing calcium to 1.75 and 2 mM. We also tested the group where calcium concentration was changed from 1.25 to 1.75 mM in the same heart (1.25→1.75). Additonally, a reduced magnesium concentration was tested (0.6 mM N=5 and 0.75 mM N=5; referred to as the “low Mg” group).

*Rat plasma collection*

To obtain samples for spectrophotometry and ISE measurements, mixed rat blood was collected immediately after decapitation into heparin coated vials and plasma was separated by the centrifugation. Hemolysis is a risk during the sample collection, which is why we are reporting results as median ± Q1,Q3, rather than means, which would have been affected by the hemolytic samples.

*Spectrophotometry (measurements of concentrations of calcium, magnesium, albumin and phosphorus)*

Calcium levels were measured using Cresolphtalein and Arsenazo reagents. Cresolphalein was analyzed at the recommended 560 nm. Arsenazo was analyzed at 670 nm (which is within the tolerance limits) because of unknown interference observed at the recommended 650 nm. Magnesium was measured using the Xilidyl blue at the recommended 520 nm. Albumin levels were measured using the Bromocresol green at the recommended 630 nm. Total protein was measured using the biuret reaction at the recommended 545 nm. Phosphorus was measured by the Phosphomolybdate reaction at the recommended 340 nm. All reagents were made by Biosystems (distributed locally by JK Trading).

Samples and reagents were mixed in Eppendorf tubes following the instructions (provided by Biosystems) then pipetted into plates and analyzed at the Thermoscientific Multiskan Sky and Tecan Genios spectrophotometers (choice was determined by the capabilities of each machine and by the currently available staff from the respective department). Samples were analyzed as duplicates to limit the impact of adverse effects, such as a hemolysis during sample collection. Storage conditions were followed according to the instructions of use of the respective reagent kit. Calibration was obtained by using the commercially available bovine or human serum calibrator (Biosystems) or by using the water calibrator included in the reagent kits (calibrators used are mentioned with each individual result).

*Calcium electrode measurements (activity of calcium ions in rat plasma and artificial solutions).*

Calcium activity was measured using combined calcium ion-selective electrode (Theta 90, local distributor Verkon). The electrode was kept submerged in the samples for at least 15 minutes to obtain stable mV values. 20 animals were used for the determination of the calcium activity in rat plasma. Because it was impossible to adhere to the anaerobic collection of rat plasma in our design, and we are of course aware, that pH affects ionized calcium, samples of rat plasma were titrated with HCl (3.5%) to obtain pH ~7.4 before the actual measurement. Dilution was negligible (~13 ul into >1 ml sample). The electrode was connected to a Jenway 3505 pH meter (impedance is needed) and its analog outputs were connected to Powerlab (LabChart software) and recorded digitally. Triangular (Barlett) window smoothing (window width 999 samples) was applied to process the raw data into final results.

*Artificial solutions*

To evaluate the effect of each individual component of the K-H solution, solutions of these components in NaCl were prepared and measured (NaCl was used to compensate for the Debye-Hückel principle, we did our best to approximate ionic strengths, and when in doubt, at least we have measured osmolality). The effect of albumin was assessed using the bovine serum albumin (BSA, P-lab) and expected value was approximated based on the data from this study (Buckley and Russell, 1988). The effect of the Debye-Hückel principle was approximated using regression from the data available in the documentation to the Theta 90 calcium electrode (assuming full dissociation of all chemicals in the K-H solution). The activity of calcium complexes was obtained by titrating the calcium in distilled water with oxalate and citrate. Osmolality values for the mannitol and glucose was approximated from the data available in the documentation to infusions and, when needed, confirmed by the osmometer. To obtain pH changes independent of bicarbonate, titration with NaOH and HCl were used. The same saturation as with the Langendorff experiments (95% O_2_ / 5% CO_2_) was used (solutions are *not* saturated, unless indicated otherwise). Individual artificial solutions were compared to controls from the same day. The precise composition of each solution is available in the table 1, and step-by-step calculations are available in the Figshare repository (DOI:10.6084/m9.figshare.28368329) All chemical substances (NaCl, KCl, KH_2_PO_4_, NaHCO_3_, glucose, mannitol, HCl, NaOH, oxalate, and citrate) were new and were obtained from Penta Chemicals. We have numbered our artificial solutions in the table 1 and are using these numbers for easy reference.

We have decided to prove the actual effect of chemicals by testing the dose-dependent relationship. Two doses of chemicals are tested: a “low” dose (N=1), corresponding to the concentration found in the standard K-H solution, and a “high” dose (N=1), which is triple that amount (in one case we also have a “moderate” value, which is double the K-H concentration). If the “high” result is “borderline”, it doesn’t make any sense to continue that investigation, as even if that was to be the true mechanism, it would not be biologically relevant anyway (in the “low” concentration). Because these experiments were performed using chemically defined artificial solutions without biological variability, a single measurement was considered sufficient to demonstrate the physicochemical effect under each condition. Rat plasma samples (N=20) were evaluated using standard statistics.

We have chosen to report our results as mV (the data obtained cast doubt on the accuracy of possible calibrations). K-H and NaCl calibration curves were obtained. Because the electrode does not give consistent voltages during measurements at different times, a following correction was applied: (C_1.25_+((M-D_1.25_)/(D_1.75_-D_1.25_)*(C_1.75_-C_1.25_))); M=measured voltage; D=calibration on the measurement day at respective calcium levels; C=calibration from the original calibration curve. This corrected voltage value was then converted to concentration using regression from the respective calibration curve. Readers should note that the relationship between mV and calcium concentration is logarithmic, not linear, when interpreting individual mV values.

*pH measurements*

pH was measured using pH meter Edge (Hanna instruments) with a combined pH electrode or by Theta 90 combined pH electrode connected to the PowerLab via Jenway 3505 pH meter. Calibration buffers (pH 4, 7 and 10) were sourced from P-lab and Hanna Instruments. The compositions of the individual solutions are available in table 1 (the solutions are numbered for easy reference). The results were either photographed (Hanna) or recorded in LabChart (Theta 90).

*Osmolality measurements*

Osmolality of rat plasma and selected artificial solutions was measured using the Osmomat 3000 basic (Gonotec). The results were photographed.

| Table 1: Composition of the solutions for the ISE measurements (mM unless stated otherwise), (t) = titration grams are per 100 ml | | | | | | | | | |
| --- | --- | --- | --- | --- | --- | --- | --- | --- | --- |
| Sol. No. | CaCl_2_ | NaCl | KCl | KH_2_PO_4_ | NaHCO_3_ | Glucose | Mannit. | MgSO_4_ | Other |
| 1 | 1.25 | - | - | - | - | - | - | - | - |
| 2 | 1.25 | 145 | - | - | - | - | - | - | - |
| 3 | 1.25 | 125 | - | - | 20 | - | - | - | - |
| 4 | 1.25 | 85 | - | - | 60 | - | - | - | - |
| 5 | 1.25 | 139.4 | - | - | - | - | 0.02 g | - | - |
| 6 | 1.25 | 128.2 | - | - | - | - | 0.06 g | - | - |
| 7 | 1.25 | 141.1 | - | - | - | 7 | - | - | - |
| 8 | 1.25 | 133.3 | - | - | - | 21 | - | - | - |
| 9 | 1.25 | 143.8 | - | 1.2 | - | - | - | - | - |
| 10 | 1.25 | 141.4 | - | 3.6 | - | - | - | - | - |
| 11 | 1.25 | 143.8 | - | - | - | - | - | 1.2 | - |
| 12 | 1.25 | 141.4 | - | - | - | - | - | 3.6 | - |
| 13 | 1.25 | 119 | 4.7 | 1.2 | 20 | 7 | 0.02 g | 1.2 | - |
| 14 | 1.25 | 119 | 4.7 | 1.2 | 25 | 7 | 0.02 g | 1.2 | - |
| 15 | 1.25 | 145 | - | - | - | - | - | - | HCl (t) pH = 5.5 |
| 16 | 1.25 | 145 | - | - | - | - | - | - | NaOH (t) pH = 8.5 |
| 17 | 1.25 | 141.4 | - | 3.6 | - | - | - | - | NaOH (t) pH = 7.4 |
| 18 | 1.25 | 141.4 | - | 3.6 | - | - | - | - | NaOH (t) pH = 8.5 |
| 19 | 2.5 | 137.5 | - | - | - | - | - | - | Albumin 4 g |
| 20 | 2.5 | 137.5 | - | - | - | - | - | - | Albumin 4 g  NaOH (t) pH = 7.4 |
| 21 | 2.5 | 117.5 | - | - | 20 | - | - | - | Albumin 4 g |
| 22 | 2.5 | 97.5 | - | - | 40 | - | - | - | Albumin 4 g |
| 23 | 2.5 | 77.5 | - | - | 60 | - | - | - | Albumin 4 g |
| 24 | 2.5 | 137.5 | - | - | - | - | - | - | Albumin 4 g  NaOH (t) pH = (23) |
| 25 | 2.5 | 137.5 | - | - | - | - | - | - | Albumin 0.75 g  HCl (t) pH = 6.5 |
| 26 | 2.5 | 137.5 | - | - | - | - | - | - | HCl volume = No. 24 |
| 27 | 2 | 108.5 | 4.5 | 1.2 | 25 | - | - | 0.8 | Albumin 4 g  “artificial human” |
| 28 | 2.4 | 115.5 | 4.5 | 1.2 | 30 | - | - | 0.8 | Albumin 2.5 g  “artificial rat” |
| 29 | 1.75 | 77.5 | - | - | 60 | - | - | - | Albumin 4 g |
| 30 | 1.75 | 137.5 | - | - | - | - | - | - | Albumin 4 g  NaOH (t) pH = (29) |
| 31 | 1.75 | 85 | - | - | 60 | - | - | - | - |
| 32 | 1.75 | 112.5 | - | - | 25 | - | - | - | Albumin 4 g |
| 33 | 1.75 | 116.25 | - | - | 25 | - | - | - | Albumin 2 g |
| 34 | 1.75 | 120 | - | - | 25 | - | - | - | - |

Notes: Calibration solutions (K-H, NaCl) were obtained by changing CaCl_2_ without any further adjustments. Titrations of citrate/oxalate were performed in distilled water. All solutions were not O_2_/CO_2_ saturated unless stated otherwise.

*Statistical analysis*

Recovery of LVDP

The results are expressed as means ± SD. Each observation was obtained from at least nine heart preparations in each group. Recovery of LVDP values were evaluated by one-way ANOVA followed by the Student-Newman-Keuls test. Differences in variances between conditions with and without preload were assessed with a location-scale model, a likelihood ratio test of a model with fixed variance against a model with variance depending on preload was performed. Presence of interactions was tested with a likelihood-ratio test.

TTC staining

Results of TTC staining were also evaluated by one-way ANOVA followed by the Student-Newman-Keuls test.

Statistical analysis was performed in R (R Core Team, 2023) using the package agricolae (De Mendiburu and Simon, 2015) and gamlss (Rigby and Stasinopoulos, 2005). The figures were created using GraphPad Prism 6.07 for Windows (Graph Pad Software, La Jolla California USA). The results were considered statistically significant when p<0.05. The code and data for the statistical analysis are available in the Figshare repository (DOI:10.6084/m9.figshare.28368329).

AZAR, T. A., SHARP, J. L. & LAWSON, D. M. 2012. Effects of cage enrichment on heart rate, blood pressure, and activity of female Sprague-Dawley and spontaneously hypertensive rats at rest and after acute challenges. *J Am Assoc Lab Anim Sci,* 51**,** 339-44.

BUCKLEY, B. M. & RUSSELL, L. J. 1988. The measurement of ionised calcium in blood plasma. *Ann Clin Biochem,* 25 ( Pt 5)**,** 447-65.

DE MENDIBURU, F. & SIMON, R. 2015. Agricolae - Ten years of an open source statistical tool for experiments in breeding, agriculture and biology. *PeerJ PrePrints,* 3:e1404v1.

DOUL, J., MINARIKOVA, M., CHARVATOVA, Z. & MAXOVA, H. 2024. Nitric oxide is involved in the cardioprotection of neonatal rat hearts, but not in neonatal ischemic postconditioning. *Physiol Rep,* 12**,** e16147.

R CORE TEAM 2023. R: A Language and Environment for Statistical Computing. R Foundation for Statistical Computing, Vienna, Austria. <https://[www.R-project.org/>](http://www.R-project.org/%3e).

RIGBY, R. A. & STASINOPOULOS, D. M. 2005. Generalized additive models for location, scale and shape (with discussion). *Appl. Statist.,* 54**,** 507-554.

SCHRECKENBERG, R., MAIER, T. & SCHLUTER, K. D. 2009. Post-conditioning restores pre-ischaemic receptor coupling in rat isolated hearts. *Br J Pharmacol,* 156**,** 901-8.
